# Omental patch repair of large perforated peptic ulcers ≥25 mm is associated with higher leak rate

**Published:** 2021-11-29

**Authors:** Yi Liang Wang, Xue Wei Chan, Kai Siang Chan, Vishal G. Shelat

**Affiliations:** ^1^Department of General Surgery, Khoo Teck Puat Hospital, Singapore; ^2^Department of General Surgery, Tan Tock Seng Hospital, Singapore

**Keywords:** peptic ulcer, perforated ulcer, gastrectomy, omental patch, ulcer size

## Abstract

**Background and Aim:**

Omental patch repair is the present gold-standard technique for patients with perforated peptic ulcers (PPUs). Data are lacking regarding the safe ulcer size for omental patch repair leak (OPL). We analyze our experience in managing PPU to identify an ulcer size cut-off for predicting OPL.

**Methods:**

Patients who had undergone omental patch repair for PPU between Jan 2004 and Apr 2016 were included. Demographic data, the American Society of Anesthesiologists score, ulcer size, operative approach, post-operative complications, and length of stay were recorded. OPL, intra-abdominal collection, repeat surgery, and 30-day mortality were recorded. The relationship between ulcer size, pre-operative characteristics, and OPL were investigated with univariate and multivariate logistic regression. Receiver operating characteristic curve analysis derived the ulcer size cut-off to predict OPL. In addition, we analyzed if ulcer size predicted mortality or malignancy.

**Results:**

Six hundred and ninety patients with a mean age of 55.1 years (range 16-94) were managed for PPU during the study period. Free air on X-ray was evident in 417 (60.4%) patients. Mean ulcer size was 7.8 mm (range 1-50). OPL occurred in 15 patients (2.2%) and 30-day mortality was 7.4% (*n*=51). Multivariate analysis found ulcer size increase of 10 mm (OR 3.30, 95% CI 1.81-6.02, *P*<0.001) predicted increased risk of OPL. At 25 mm cut-off, sensitivity was 26.7%, specificity was 97.2%, positive likelihood ratio was 9.47, and negative likelihood ratio was 0.76 for OPL.

**Conclusion:**

Ulcer size increase in 10 mm increases leak rate by 3.3 times. Ulcer size ≥25 mm predicts OPL.

**Relevance for Patients:**

Increased risk of OPL for ≥25 mm warrants need for close post-operative monitoring and lowers threshold for investigations in event of clinical deterioration. Decision for omental patch repair versus gastrectomy however should not be based on ulcer size alone.

## 1. Introduction

The first series of perforated peptic ulcers (PPUs) was first published by Edward Crisp in 1843 [1]. PPU is a surgical emergency [1]. Omental patch repair, first described by Cellan-Jones in 1929 and later modified by Graham, is a safe and feasible treatment option and is established as the gold standard in the management of PPU [2-4]. Earlier studies have reported mortality rates as high as 40% [5,6]. With advances in critical care and surgical technique, the surgical outcomes of PPU have improved.

Omental patch repair leak (OPL) is a feared complication and a significant cause of mortality. In a study including 422 patients with PPU and treated by omental patch repair, Maghsoudi and Ghaffari reported a 4% (*n*=17) leak rate with 29.4% mortality in patients who experienced a leak [7]. In another study including 119 patients with perforated duodenal ulcer and 7.6% (*n*=9) leak rate, Kumar *et al*. reported 55.6% mortality in patients who experienced a leak, with ulcer size being an independent predictor of leak [8]. Since ulcer size is an established risk factor of OPL, caution is needed in treating large PPUs managed with omental patch repair. In patients with large gastric or duodenal ulcers, antrectomy, distal gastrectomy, jejunal serosal patch, or Roux-en-Y duodenojejunostomy are often advocated [9]. In one study, approximately 10% of patients were managed with gastric resections due to large ulcer size or suspected malignancy [10].

The widespread availability of proton pump inhibitor therapy, *Helicobacter pylori* eradication, and advances in endoscopy technique have streamlined the clinical management of gastric and duodenal ulcers, and many authors report unified outcomes of gastric and duodenal ulcers [9]. Since ulcer size is only known during surgery, the surgeon has to be prepared and well-equipped with the expertise to tailor the conduct of surgery. Intra-operative risk assessment based on ulcer size may aid clinical decision making, for example, in deciding when to call for additional help or when to consider a gastric resection. Once a patient is predicted to have an increased risk of morbidity, post-operative care can be tailored to increase vigilance in monitoring for complications. Our study aims to identify the upper limit of ulcer size beyond which omental patch repair has a high risk of OPL.

## 2. Methods

This is a retrospective study of all patients with PPU treated with omental patch repair at a university-affiliated academic hospital between Jan 2004 and Apr 2016. Patients were identified by an electronic search of diagnosis and operative codes. Patients who were managed non-operatively, had undergone alternative modalities of surgery (e.g., serosal patch repair [*n*=2], ulcerectomy with pyloric diversion [*n*=3] or gastrectomy [*n*=75]), or who had a perforation of other organs were excluded from the study. Details with regards to diagnosis coding and the institutional algorithm in managing PPU are previously reported [11].

### 2.1. Study variables and outcomes

Demographic data, including age, gender, co-morbidities, and duration of symptoms, were collected. Lifestyle-related risk factors (e.g., smoking) and medication history (e.g., non-steroidal anti-inflammatory drugs [NSAIDs] or steroids) were analyzed. The presence of circulatory shock (defined as systolic blood pressure <90 mmHg on presentation), the American Society of Anesthesiologists (ASA) score, operative findings, and duration of operation were recorded. Intra-operative measurement of ulcer size with a sterile paper ruler was routinely performed. Post-operative morbidity included intra-abdominal collection, OPL and need for reoperation. OPL was defined as any post-operative drainage effluent with bilious or gastrointestinal contents or detected on imaging in patients with deviation from expected recovery trajectory or was established at reoperation. 30-day mortality was defined as death within 30 days following surgery.

### 2.2. Pre-operative management and surgical technique

On suspicion of perforated ulcer, all patients were given intravenous amoxicillin-clavulanate 1.2 g and stat dose of gentamicin at 5 mg/kg in accordance to local antibiotogram. Patients with hemodynamic instability were managed according to sepsis guidelines. Nasogastric tube (NGT) was routinely placed pre- or intra-operatively for gastric decompression. Our institution triages the urgency of surgery using a central anesthetist led triage system of P0, P1, P2, P3A, P3B, and P4 categories. P0 indicates life-threatening disease which requires immediate surgery, while P4 indicates stable disease which does not require urgent surgery. Time to surgery for P0, P1, P2, P3A, P3B and P4 are as follows: immediate, within 1 h, within 4 h, within 8 h, within 12 h and within 24 h, respectively. All cases of PPU are triaged as P2 by default, with variability allowances for reasonable clinical judgment in selected patients.

Surgery was done under general anesthesia for all patients. Two types of repair techniques were used for patients included in this study: open suture omental patch repair, and laparoscopic suture omental patch repair. Sutureless repair was not used in view of the lack of high-quality generalizable evidence [9]. Choice of technique was based on surgeon’s discretion and expertise. Laparoscopic approach was performed by a specialist surgeon, or a trainee under direct supervision of the specialist. Patients with Boey score ≤1, ulcer size ≤10 mm, no previous abdominal surgery, and hemodynamically stability were considered suitable for laparoscopic repair [12]. Diagnostic exploration and warm saline irrigation was done for all cases. Intra-operatively, biopsy was taken at discretion of the operating surgeon; duodenal ulcers have very low risk of gastric cancer [13]. Peritoneal fluid was sent for fluid culture at discretion of the operating surgeon. Polydioxanone 2/0 suture was routinely used for omental patch repair. Choice of omental patch repair alone versus primary repair with omental patch buttress was left to surgeon’s discretion. Almost all patients managed with open laparotomy underwent omental patch repair alone without primary closure of ulcer, that is, Graham’s patch repair. In majority of instances of laparoscopic PPU repair, the perforation was first suture closed and then omental patch was placed as a buttress, that is, Cellan-Jones repair. Our unit typically does a 3-port laparoscopy, and it is relatively difficult to keep the omentum in position while performing intra-corporeal suturing. Further, Graham’s patch requires that suture material is left *in situ* until the omental patch is placed over the ulcer defect, with subsequent tying of knots. During laparoscopic surgery, this leads to “suture traffic” in the abdominal cavity and could compromise the precision of knotting.

A closed suction Jackson-Pratt^®^ drain was routinely placed intra-operatively with intent to monitor for early detection of post-operative leak and prevention of intra-abdominal collection. Drains were removed when the effluent was below 50 ml/day on two consecutive days, or at surgeon’s discretion. NGT was removed according to clinical judgment which was determined by history, physical examination, and chart reviews. History of passage of flatus and bowel opening, soft and non-distended abdomen with active bowel peristaltic sounds, and <500 ml output over the past 24-h period would prompt for NGT removal consideration. In alignment with enhanced recovery after surgery principles, every morning rounds a conscious deliberation was done if NGT withdrawal is safe.

Patients who had juxtapyloric or duodenal ulcers were treated empirically with *H. pylori* eradication triple therapy upon discharge [14,15]. In patients with deviation from expected recovery path, as for example, pyrexia, hemodynamic instability, or evolving abdominal symptoms or signs, an urgent computed tomography (CT) scan of the abdomen and pelvis with oral and intravenous contrast was performed to diagnose leak as well as guide management. In patients with obvious bilious fluid or gastrointestinal contents in the drain tube, a CT scan was still performed to guide further management, as for example if placement of image guided percutaneous drains was warranted for source control.

### 2.3. Statistical analysis

Data analysis was performed with SPSS Statistics 26 (IBM, New York, USA). Univariate logistic regression was used to evaluate the effect of pre-operative factors and that of increasing ulcer size on the probability of OPL. Statistically significant associations (*P*<0.05) were further evaluated with multivariate logistic regression to identify factors independently associated with OPL. Receiver-operating characteristics (ROC) analysis was used to delineate the relationship between increasing ulcer size and OPL, and to identify an optimal size cut-off for omental patch repair, following which, risk of OPL is deemed to be high. ROC analysis was also performed to evaluate if ulcer size predicted mortality and malignancy.

## 3. Results

During the study duration, 690 patients with a mean age of 55.1 years (range 16-94) were treated with omental patch repair. The majority of patients were male (*n*=511, 74.1%). Table 1 summarises the demographics of the study population. 663 (96.1%) were treated with open surgery. The mean ulcer size was 7.8 mm (range 1-50). The mean operative duration was 88.0 min (range 30-325). Peri-operative outcomes are summarized in Table 2.

**Table 1:** Demographic and clinical profile of patients with perforated peptic ulcer treated with omental patch repair

	*n*=690 (%)
Mean age, years	55.1 (16-94)
Risk factors	
Smoking	221 (32)
Previous peptic ulcer	52 (7.5)
NSAID use	33 (4.8)
Steroid use	22 (3.2)
Co-morbidities	
Diabetes mellitus	81 (11.7)
Ischemic heart disease	34 (4.9)
Chronic kidney disease	23 (3.3)
Chronic obstructive pulmonary disease	16 (2.3)
Investigations	
Hemoglobin, g/dL	13.5 (4.8-20.4)
White blood cell count, 10^9^/L	12.9 (1.2-68.9)
Albumin, g/L	34.8 (10-59)
Creatinine, µmol/L	110 (24-522)
Free air on chest radiograph	417 (60.4)
Perforation on computed tomography scan	322 (98.8)*
Shock^#^	41 (5.9)
Delayed presentation of >24 h	319 (46.2)
Mortality prediction models	
ASA score, median (IQR)	2 (2-3)
Boey score, median (range)	1 (0-3)
Mannheim Peritonitis Index, mean (range)	15.0 (0-41)

All continuous variables are expressed as mean (range) unless stated otherwise.

* Value in parenthesis is expressed as percentage of patients who had computed tomography scan (*n*=326).

# Shock is defined as a systolic blood pressure of <90mmHg during triage on presentation. ASA: American Society of Anesthesiologists, NSAID: Non-steroidal anti-inflammatory drug

**Table 2:** Peri-operative outcomes patients with perforated peptic ulcer managed with omental patch repair

	*n*=690
Ulcer size, mm	7.8 (1-50)
Location of ulcer	
Stomach	265 (38.4)
Duodenum	420 (60.9)
Jejunum	5 (0.7)
Duration of operation, min	88 (30-325)
Length of stay, median, days (IQR)	7 (5-11)
Total parenteral nutrition use, *n*	61 (8.8)
Blood transfusion, *n*	61 (8.8)
Empiric *Helicobacter pylori* eradication, *n*	448 (64.9)
Histology taken, *n*	214 (31.0)
Malignancy, *n*	5 (2.3)*
ICU admission, *n*	116 (16.8)
Post-operative morbidity	
Intra-abdominal collection	37 (5.4)
Omental patch repair leak	15 (2.2)
Re-operation	12 (1.7)
30-day mortality	51 (7.4)

All continuous variables are expressed as mean (range) unless otherwise stated. All categorical variables are expressed as n (%) unless otherwise stated.

* Expressed as a percentage of patients who had histology taken. IQR: Interquartile range, ICU: Intensive care unit

Pre-operative factors and ulcer size were evaluated for an association with OPL with univariate logistic regression, as detailed in Table 3. Female gender, increasing age, NSAID use, glucocorticoid use, diabetes mellitus, chronic kidney disease, decreasing hemoglobin, increasing urea, increasing ASA score, increasing ulcer size, increasing Boey’s score, and Mannheim Peritonitis Index were significantly associated with an increased risk of OPL on univariate analysis. On multivariate analysis, however, only increasing ulcer size was independently associated with increased odds of OPL (OR 3.30, 95% CI: 1.81-6.02, *P*<0.001).

**Table 3:** Risk factors for omental patch repair leak

Risk factor	Univariate analysis		Multivariate analysis	
	
	Odds ratio (95% CI)	*P*-value	Odds ratio (95% CI)	*P*-value
Gender (female)	4.54 (1.56-12.5)	0.005	2.08 (0.49-8.92)	0.322
Increasing age (per year)	1.05 (1.02-1.09)	0.003	1.01 (0.96-1.06)	0.723
Smoking	0.15 (0.02-1.13)	0.066	-	-
Previous peptic ulcer	1.92 (0.42-8.76)	0.40	-	-
NSAID use	5.28 1.44-20.1)	0.012	5.45 (0.93-31.8)	0.060
Steroid use	13.3 (3.86-45.7)	<0.001	6.96 (0.92-52.6)	0.060
Diabetes mellitus	5.33 (1.85-15.4)	0.002	3.10 (0.71-13.5)	0.133
Ischaemic heart disease	1.39 (0.18-10.8)	0.754	-	-
Chronic kidney disease	8.19 (2.14-31.3)	0.002	5.46 (0.78-38.5)	0.088
Chronic obstructive pulmonary disease	3.14 (0.39-25.5)	0.283	-	-
Anemia (per 1 g/dL decline in hemoglobin)	1.21 (1.03-1.43)	0.023	0.94 (0.76-1.16)	0.556
Leukocytosis, (per unit 10^9^/L increase in white cell count)	0.92 (0.83-1.02)	0.122	-	-
Hypoalbuminemia (per 1g/dL decrease in albumin)	1.05 (0.99-1.11)	0.117	-	-
Creatinine (per mmol/L increase)	1.01 (1.00-1.01)	0.056	-	-
Urea (per mmol/L increase)	1.04 (1.01-1.09)	0.029	0.95 (0.84-1.07)	0.406
ASA score	3.30 (1.76-6.17)	<0.001	1.21 (0.51-2.84)	0.828
Increasing ulcer size (per 10 mm increase)	2.87 (1.89-4.35)	<0.001	3.30 (1.81-6.02)	**< 0.001**
Delayed presentation >24 h	2.37 (0.80-6.99)	0.119	-	-
Shock: admission SBP <90 mmHg	1.00 (0.99-1.01)	0.929	-	-
Boey’s Score (per 1 point increase)	2.58 (1.44-4.63)	0.001	0.90 (0.32-2.56)	0.849
Mannheim Peritonitis Index (per 1 point increase)	1.15 (1.08-1.23)	<0.001	1.06 (0.95-1.19)	0.291
Laparoscopic access	NA*	NA*	-	-

* Analysis not possible as no leaks occurred among laparoscopic cases. ASA: American Society of Anesthesiologists, NSAID: Non-steroidal anti-inflammatory drug, SBP: Systolic blood pressure

**Figure 1:**
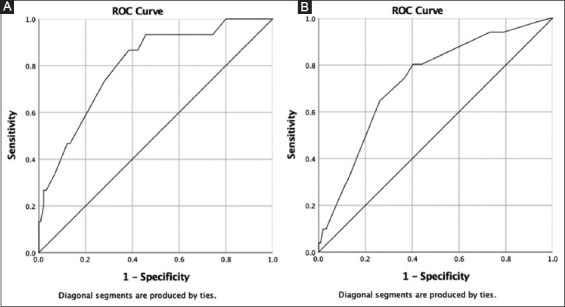
Receiver-operating characteristics curve correlating ulcer size (mm) with (A) omental patch repair leak and (B) 30-day mortality.

ROC analysis correlating ulcer size with OPL yielded an area under curve (AUC) of 0.802 (95% CI: 0.697, 0.906) (Figure 1A). At 25 mm ulcer size cut off, incidence of leak was 17.4% and 1.7% for ulcer size ≥25 mm and <25 mm, respectively, with sensitivity of 26.7%, specificity of 97.2%, positive likelihood ratio (LR) of 9.47 and negative LR of 0.76 (Table 4). ROC analysis correlating ulcer size with mortality yielded an AUC of 0.731 (95% CI: 0.66, 0.80) indicating fair performance (Figure 1B). At 25 mm ulcer size cut off, incidence of mortality was 21.7% and 6.9% for ulcer size ≥25 mm and <25 mm, respectively, with sensitivity of 9.8%, specificity of 97.2%, positive LR of 3.48 and negative LR of 0.93 (Table 5). We also additionally performed ROC analysis correlating ulcer size with malignancy, yielding AUC of 0.662 (95% CI 0.409, 0.914), indicating poor performance.

**Table 4:** Ulcer size cut-offs for omental patch repair with respective leak rate, sensitivity, specificity, positive and negative LR predictive of omental patch repair leak

Ulcer size cut-off, mm	Leak rate if ulcer size ≥ specified cut-off (%)	Leak rate if ulcer size < specified cut-off (%)	Sensitivity (%)	Specificity (%)	Positive LR	Negative LR
15	7/89 (7.9)	8/601 (1.3)	46.7	88.0	3.88	0.61
20	5/50 (10.0)	10/640 (1.6)	33.3	93.3	5.00	0.71
25	4/23 (17.4)	11/667 (1.7)	26.7	97.2	9.47	0.76
30	3/17 (17.7)	12/673 (1.8)	20.0	97.9	9.64	0.82

LR: Likelihood ratio

**Table 5:** Ulcer size cut-offs for omental patch repair with respective mortality rate, sensitivity specificity, positive and negative LR predictive of 30-day mortality

Ulcer size cut-off, mm	Mortality rate if ulcer size ≥ specified cut-off (%)	Mortality rate if ulcer size < specified cut-off (%)	Sensitivity (%)	Specificity (%)	Positive LR	Negative LR
15	15/89 (16.9)	36/601 (6.0)	29.4	88.4	2.54	0.80
20	9/50 (18.0)	42/640 (6.6)	17.7	93.6	2.75	0.88
25	5/23 (21.7)	46/667 (6.9)	9.8	97.2	3.48	0.93
30	5/17 (29.4)	46/673 (6.8)	9.8	98.1	5.21	0.92

LR: Likelihood ratio

## 4. Discussion

Peptic ulcer disease occurs due to an imbalance between mucosal defensive factors (such as the mucus-bicarbonate layer, prostaglandins, cellular regeneration, and healthy mucosal blood flow) and aggravating factors (such as *H. pylori* infection, acidity, pepsin, smoking, ethanol, NSAIDs, and steroids) [16]. Peptic ulcer disease is common, and hospitalization rates as high as 160-210 cases per 100,000 person-years have been reported [16,17]. PPU is a complication of peptic ulcer disease and requires emergency surgical intervention. Omental patch repair is the standard surgical technique for almost a century as it is safe with acceptable peri-operative risks [3,4,18,19] In this single-center retrospective study including a large sample of PPU patients, we have shown that increasing ulcer size is significantly associated with a higher risk of OPL, and ulcer size ≥25 mm strongly predicts leak rate. Kumar *et al*. identified ulcer size ≥5 mm as predictor of leak following omental patch repair of perforated duodenal ulcer [8]. Lee *et al*. similarly demonstrated that ulcer size >5 mm is associated with significantly higher leak rate following laparoscopic fibrin glue repair of PPU (leak rate for ulcer size >5 mm: 29%, ≤5 mm: 10%) [20]. They, however, failed to demonstrated any difference in leak rate for laparoscopic suture repair, though this was attributed to small sample size. The recently published the World Society of Emergency Surgery (WSES) 2020 guidelines suggest ulcer size <20 mm to be managed with primary repair with or without omental patch, in view of low post-operative leak rates reported up to 2 cm [21,22]. However, there is no standardized cut-off for ulcer size to predict high leak rates. Hence, this study aims to address this knowledge gap.

Ulcer size determines the choice of technique. Traditionally, large ulcers are managed by alternative techniques. In a retrospective study including 162 patients with perforated duodenal ulcers, Gupta et al. have shown that small (<1 cm size, *n*=122) and large (1-3 cm size, *n*=38) ulcers can be safely managed with an omental patch, but giant (>3 cm size, *n*=2) ulcers are associated with a high risk of leak [22]. Sharma *et al*. defined ulcers >2.5 cm in size as a giant and advocate that such ulcers should not be repaired by simple techniques [23]. They report a series of seven PPU patients with giant ulcers who were treated with a free omental plug with excellent outcomes. The definition of giant ulcer is inconsistent in literature, with variable definitions of ulcers being larger than 2-3 cm in size [9,24,25]. It is also unclear if gastric resection procedures for giant ulcers reduce the morbidity burden [26]. In a study including 62 PPU patients with gastric resections, Seow *et al*. have reported a malignancy risk of 3%, morbidity of 27.7%, and mortality of 24.2% [10], resulting in the conundrum of the choice of omental patch repair versus gastric resection: omental patch repair for large ulcers has high leak rates, yet gastric resections are associated with inferior outcomes. A recent retrospective observational study by Chan *et al*. in 2019 on 110 patients with PPU demonstrated that the outcomes of omental patch repair are comparable to gastric resection in patients with PPU of ≥20 mm [26]. In cases of large gastric ulcers with suspicion of malignancy, resection with intra-operative frozen pathologic examination is proposed. For large duodenal ulcers, resection or repair with or without pyloric exclusion and external bile drainage is advised. These are, however, grade 2D recommendations which are weak recommendations based on very low-quality evidence [21]. Therefore, more evidence is required to guide the modality of treatment based on ulcer size. To date, however, there is no literature detailing the optimal ulcer size cut-off for safe omental patch repair. While we obtained similar positive LR for cut-off of 25 mm and 30 mm, we propose the use of 25 mm as a cut-off in clinical practice, as a liberal policy of gastric resections is also fraught with high morbidity and mortality [10,27].

Our study reported low incidence of malignancy of 2.3% compared to existing literature. Hodnett *et al*. reported 7.6% incidence of malignancy in 202 patients with perforated gastric ulcer [28], while a review by Roviello *et al*. reported 10-16% incidence of gastric cancer in patients with gastric perforation [29]. Perforation of gastric cancer occurs more frequently at advanced stage of disease [30]. In addition, our data reported location of ulcer in the stomach, duodenum and jejunum. The incidence of gastric cancer is significantly low in duodenal ulcers [13]. Low malignancy rates reported by our institution may be attributed due to different patient demographics, increased population willingness to learn about *H. pylori* infection, and liberal use of esophagogastroduodenoscopy in patients with epigastric pain symptoms [31]. In local context, majority of gastric cancers are identified through esophagogastroduodenoscopy done for evaluation of patients with epigastric pain, constitutional symptoms and anemia.

Our series also reported low 30-day mortality of 7.4% which is consistent with existing literature ranging 1.3% to 20% [9,32]. Delay in surgery has been associated with higher mortality and has been postulated to be due to the extent of peritoneal contamination [33]. A large nationwide cohort study by Boyd-Carson *et al*. in 2020 on 3809 patients with PPU who underwent emergency laparotomy within 24 h showed an adjusted 4% increase in mortality per every hour delay to surgery, and an adjusted 6% increase in mortality per every hour delay in patients with shock [34]. Median time to surgery from admission in their study was 7.5 h (interquartile range 5-11.6 h) [34]. Svanes *et al*. reported marked delay of ≥12 h to surgery resulted in higher mortality (22.8% vs. 5.9%, *P*<0.001), post-operative complications (48.6% vs. 24.6%, *P*<0.001) and prolonged stay >14 days (33.8% vs. 20.3%, *P*=0.001) in ≥50-years-old patients [35]. Even though we did not collect data on the time to surgery, our institution triages PPU as P2 category (to be done within 4 h) as a default. Thus, majority of our PPU patients are operated within 12 h of diagnosis. This may explain our low mortality rate. The use of ulcer size cut-off of 25 mm is, however, not a good predictor of mortality, with AUC of 0.731 indicating fair performance. A positive LR of above 10 is considered to provide strong evidence to predict risk [27]; our study obtained a positive LR of 3.48. We propose that ulcer size of ≥25 mm should be used to predict risk of leak, but not 30-day mortality. In our opinion, mortality risk in patients with sepsis is also determined by underlying patient comorbidities (e.g., diabetes mellitus), and thus independent of ulcer size [36].

The choice of surgical access (open versus laparoscopic) has also been debated on its impact on leak rate. Laparoscopic omental patch repair is safe and feasible but was only offered to 4% (*n*=27) of our patients in our experience [18,37,38]. Patients with PPU often present after office hours with a lack of supervision, creating a barrier for adopting laparoscopic approach. A recent meta-analysis by Cirocchi et al. included eight randomized controlled trials and 615 patients (307 patients undergoing laparoscopic repair and 308 patients undergoing open repair) and concluded that laparoscopic repair was associated with less post-operative pain and surgical site infections, with comparable leak rates [39]. However, surgeon experience was only reported in one study by Lau *et al*. [40]. We report good results with no leak following laparoscopic omental patch repair, and we are unable to comment on the impact of surgical access on leak rate. This may be attributed to good selection criteria in patients who had laparoscopic repair [12]. This is in line with the WSES 2020 guidelines that laparoscopic omental patch repair should be reserved for stable patients in the context where technical expertise and equipment is available [21].

The strength of our study is the large sample of patients with duodenal and gastric ulcers and the robust electronic medical records, which provided fidelity in data collection. There are, however, some limitations of this retrospective study. Firstly, a single-centre experience makes our patient demographic and clinical profile potentially different from other institutions. Thus, the results may not be generalizable. Secondly, we did not record the cause of mortality; it is understood that mortality outcomes are not solely dependent on the development of OPL but are also influenced by other co-morbidities [36]. In our opinion, mortality outcomes are not contributed by policy of not-prescribing antifungal therapy routinely [41]. A randomized controlled trial comparing the outcomes of omental patch repair with gastric resections in large or giant ulcers is warranted. As large or giant ulcers are uncommon, a multi-centre trial is essential for adequate power. We estimate that such a trial will need to enrol 250 patients in each arm to find a 10% difference in comprehensive complication index with 80% power, two-sided alpha of 5%. Finally, we did not report on other morbidity outcomes such as surgical site infection, burst abdomen, urinary tract infections, pleuropulmonary complications, nor did we classify morbidity based on the widely accepted Clavien-Dindo classification system.

## 5. Conclusion

Omental patch repair remains the gold standard of treatment for PPU. However, our study demonstrated that increase in ulcer size is an independent predictor of OPL, with a 3.3 times increase in leak rate for every 10 mm increase in ulcer size. Ulcer size of ≥25 mm can be used as a guide in surgical practice to predict leak rate. It is, however, not a good predictor of 30-day mortality. Identification of this subgroup of patients with PPU will guide the choice of surgical procedure, and tailor post-operative care to increase vigilance in monitoring for complications.

### Conflict of Interest

The authors declare no conflicts of interest.
